# Arterial stiffness in adults with steady-state bronchiectasis: association with clinical indices and disease severity

**DOI:** 10.1186/s12931-018-0790-3

**Published:** 2018-05-09

**Authors:** Yong-hua Gao, Juan-juan Cui, Ling-yun Wang, Ke-qin Yin, Li Wang, Guo-jun Zhang, Shao-xia Liu

**Affiliations:** 1 Department of Respiratory and Critical Care Medicine, The First Affiliated Hospital of Zhengzhou University, 1 Jianshe East Road, Zhengzhou, 450052 Henan China; 2 Department of Ultrasound Medicine, The First Affiliated Hospital of Zhengzhou University, Zhengzhou, Henan China

**Keywords:** Bronchiectasis, Arterial stiffness, Pulse wave velocity, Systemic inflammation, Cardiovascular disease

## Abstract

**Background:**

Cardiovascular disease are common co-morbidities in bronchiectasis and contribute substantially to disease burden and mortality. Brachial-ankle pulse wave velocity (baPWV), a measure of arterial stiffness, has a strong predictive value for cardiovascular event. We hypothesized that baPWV would be increased in steady-state bronchiectasis patients, and correlates with the degree of systemic inflammation and disease severity assessed with *Bronchiectasis Severity Index* and FACED scores.

**Methods:**

Eighty patients with steady-state bronchiectasis and 80 age- and sex-matched controls were enrolled. BaPWV was measured as an indicator of arterial stiffness. Demographic, clinical indices, radiology, spirometry, sputum bacteriology and systemic inflammatory mediators were also assessed.

**Results:**

Bronchiectasis patients had significantly increased baPWV [median 1514 cm/s vs. 1352 cm/s, *P* = 0.0003] compared with control subjects. BaPWV significantly correlated with *Bronchiectasis Severity Index* (rho = 0.65, *P* < 0.001) and FACED (rho = 0.49, *P* < 0.001) scores. In multivariate regression analysis, age, *Pseudomonas aeruginosa* colonization, systolic blood pressure, body-mass index and exacerbation frequency in the last 12 months, but not systemic inflammatory markers, were independent factors influencing on baPWV in bronchiectasis patient after adjustment for other clinical variables. Reproducibility of baPWV measurement was good.

**Conclusion:**

Bronchiectasis patients have increased arterial stiffness compared with control subjects, which correlates with disease severity, but not systemic inflammatory markers. Age, *Pseudomonas aeruginosa* colonization, systolic blood pressure, body-mass index and exacerbation frequency in last 12 months might independently predict the severity of arterial stiffness in bronchiectasis. Therefore, arterial stiffness might have contributed to the increased risks of developing cardiovascular diseases in bronchiectasis.

**Electronic supplementary material:**

The online version of this article (10.1186/s12931-018-0790-3) contains supplementary material, which is available to authorized users.

## Background

Bronchiectasis is a chronic airway disease characterized by recurrent infection and chronic inflammation. The prevalence of bronchiectasis has been increasing over the past decade and led to substantial morbidity and mortality worldwide [1–3]. Recent studies have demonstrated that patients with bronchiectasis have increased risks of developing cardiovascular disease (CVD), and that concomitant CVD might pose a negative impact on survival [4–7]. The underlying mechanisms of elevated cardiovascular risk are still unclear, but increased systemic inflammation and frequent exacerbations due to chest infections might play a crucial role [5, 8].

Arterial stiffness reflects the decreased capability of an artery to dilate and contract in response to pressure changes. A multitude of non-invasive methods have been developed to assess arterial stiffness [9, 10]. Of these, pulse-wave velocity (PWV) is the most widely used and validated technique [10]. The carotid-femoral PWV (cfPWV) is the gold standard for assessing arterial stiffness [9, 11], but requires patients’ persistent lateral rotation of the neck and exposure of inguinal region [12]. However, brachial-ankle PWV (baPWV) has been extensively applied for assessment in East Asian countries (including China), presumably due to the greater ease and convenience over a longer arterial length, and recent evidence has shown similar usefulness of baPWV in CVD risk stratification compared with cfPWV [13].

Previous studies suggested that individuals with chronic respiratory diseases have increased arterial stiffness compared with age- and sex-matched control subjects [14–16]. This has led us to postulate that similar conclusions apply to bronchiectasis patients because they share some common biological pathways for developing CVD (i.e. hypoxia and systemic inflammation) [8, 17]. In a small, single-center study (20 patients versus 20 controls), Gale and colleagues [18] have shown that bronchiectasis patients had greater arterial stiffness, but they defined arterial stiffness by measuring aortic PWV. *Bronchiectasis Severity Index* (BSI) and FACED score have been widely used to categorize the severity of bronchiectasis [19, 20]. However, their relationship with arterial stiffness remained unclear. In addition, bronchiectasis exacerbations are common and important events related to exaggerated airway infection and systemic inflammation [21, 22], which might accelerate the process of impairment of the cardiovascular system.

Therefore, we aimed to: (1) assess arterial stiffness in bronchiectasis patients by measuring baPWV as compared with age- and sex-matched healthy controls; (2) elucidate the relationship between disease severity and arterial stiffness; and (3) determine the factors influencing on arterial stiffness in patients with bronchiectasis.

## Methods

### Study population

Eighty-nine steady-state bronchiectasis patients (defined as no major deterioration in clinical signs and symptoms within 4 weeks) aged 18 years or greater were prospectively recruited from the pulmonology clinic of The First Hospital of Zhengzhou University between January 2016 and March 2017. Patients were enrolled if they had a clinical diagnosis of bronchiectasis based on high-resolution computed tomography (HRCT) combined with a compatible history (i.e. chronic cough, sputum production or hemoptysis etc.). Subjects were excluded if they (1) had severe cardiovascular disease (i.e. uncontrolled congestive heart failure, unstable angina pectoris or myocardial infarction within 3 months); (2) had uncontrolled hypertension [systolic blood pressure (SBP) ≥160 mmHg and/or diastolic blood pressure (DBP) ≥90 mmHg]; (3) had renal or hepatic dysfunction; (4) had malignancy; (5) refused to perform baPWV measurement. Patients with traction bronchiectasis due to severe emphysema or advanced fibrosis, and uncontrolled asthma were excluded. Finally, a total of 80 subjects were included for the analysis. Meanwhile, 80 healthy subjects with normal chest X-ray and no prior history or symptoms of chronic respiratory diseases (including asthma, chronic obstructive pulmonary disease, etc.) who were individually matched with bronchiectasis patients for age and sex were recruited during the same period from the Health Check-up Center. Ethical approval was obtained by the Ethics Committee of The First Affiliated Hospital of Zhengzhou University. Written informed consent was obtained.

### History inquiry and disease severity assessment

Data collection included demographic data, anthropometric measures, smoking history, comorbidities, the number of exacerbations and hospitalizations in the previous year, current treatments. Exacerbation was defined as a significant increase in respiratory symptoms (increased cough frequency, increased sputum volume or viscosity, increased sputum purulence with or without increased wheezing, breathlessness, haemoptysis) and/or systemic upset requiring antibiotic treatment [23]. Exacerbation history was recorded according to patient recall, followed by verification of available medical charts. Chest HRCT scan within 12 months was used for radiologic scoring based on the number of bronchiectatic lobes (with the lingula being scored as a separate lobe) and the severity of bronchial dilatation (tubular: 1 point, varicose: 2 points, cystic: 3 points), with the maximal score of 18 [20]**.** Aetiology of bronchiectasis was determined as previously described [24, 25]. Cystic fibrosis and *a*1 anti-trypsin deficiency have been scarcely reported in Asian countries, therefore routine screening was not conducted [24].

Disease severity was assessed with both BSI and FACED score [19, 20], respectively. The items of BSI included age, body-mass index (BMI), exacerbation frequency, prior hospitalization, MRC dyspnea score, forced expiratory volume in one second (FEV_1_) predicted%, *Pseudomonas aeruginosa* (PA) colonization, colonization with other potentially pathogenic microorganisms (PPMs) and the number of bronchiectatic lobes, The BSI of 0–4, 5–8, and 9 or greater corresponded to mild, moderate and severe bronchiectasis, respectively. FACED score consisted of FEV_1_ predicted%, age, PA colonization, radiological extension and MRC dyspnea score. The FACED score of 0–2, 3–4, and 5–7 corresponded to mild, moderate and severe bronchiectasis, respectively.

### Laboratory assessment

Venous blood was drawn from bronchiectasis patients after fasting overnight for at least 8 h before venipuncture. Total cholesterol, triglycerides and C-reactive protein were measured by standardized and certified program using automatic biochemical analyzer (AU5800, BECKMAN COULTER, USA). Serum interleukin (IL)-6 and IL-8 were measured using commercial multiplex bead-based assay kits described previously (Bio-Plex Cytokines Assay; Bio-Rad Inc) [23]. Citrated plasma samples were analyzed for fibrinogen using the Clauss method (IL ACL Top Coagulation Analyzer; Instrumentation Laboratories, Lexington, MA).

### Sputum bacteriology and spirometry

Fresh sputum was sent for culture within 2 h after removing oral cavity debris. Hypertonic saline (3%~ 5%) induction was applied when appropriate. Colonization of PA and other PPMs was defined as sputum culture positive for two or more occasions (at 3-month interval) for the same pathogenic bacteria within one year [23].

At baseline, all steady-state bronchiectasis patients underwent spirometry (QUARK PFT, COSMED Inc., Italy) according to international guidelines [26]**.** Results were originated from three technically repeatable maneuvers, with between-maneuver variation < 5% or 200 ml in forced vital capacity (FVC) and FEV. The best FVC and FEV_1_ were reported.

#### Measurement of baPWV

A well-trained examiner (J.J.C.) measured baPWV among bronchiectasis patients using a non-invasive vascular screening device (BP-203RPE III, OMRON, Japan) according to manufacturer’s instructions. Participants have rested for at least 10 min in supine position before measurement. A plethysmographic sensor was connected to the cuffs, which was wrapped around the brachia and ankles, to record simultaneously pulse volume waveforms. The time interval (ΔT) from the wave fronts of the brachial to ankle waveforms was recorded. The length between brachium and ankle (ΔL) was adjusted for participant’s height [9]. The baPWV was calculated for each side by using the formula: baPWV = ΔL/ΔT (cm/s). The average of baPWV for both sides was used for subsequent analyses. Higher baPWV corresponded to greater arterial stiffness and cardiovascular risk [9].

Reproducibility was assessed by measurement of baPWV between baseline and 2 weeks apart (from 8 a.m. to 12 a.m.). Of the initial 20 bronchiectasis patients invited to participate the reproducibility study, two patients dropped out and one was excluded due to exacerbation during 2-week follow-up. Therefore, 17 patients (12 women and 5 men) were included in reproducibility analysis, with a mean age of 47.6 years. Notably, treatment strategies did not change at 2-week interval. The majority of patients were categorized as having moderate-to-severe bronchiectasis as assessed with the BSI, and three patients were classified as having mild bronchiectasis. Notably, patients were required to avoid physical activity, tobacco smoke for at least 12 h, and vitamin supplementation for 72 h prior to each baPWV measurement.

#### Statistical analysis

Data were presented as mean ± standard deviation (SD), median (interquartile range, IQR) or frequencies (proportion) as appropriate. Categorical variables were compared using χ^2^ tests. For continuous variables, two-group comparisons were performed using unpaired t tests or Mann-Whitney tests when appropriate. Spearman’s correlation coefficients and intraclass correlation coefficients were used to compare baPWV values at baseline versus 2-week interval. Bland-Altman plot was used to demonstrate the repeatability of baPWV over time. Univariate and multivariate linear regression analysis were used to examine factors affecting baPWV in bronchiectasis patients. Cutoffs of *P* values < 0.1 in univariate models were initially eligible in multivariate model and removed using backward selection. The correlation between baPWV and bronchiectasis severity was determined with Spearman’s rank or Pearson’s correlation coefficient when appropriate. *P* < 0.05 was considered statistically significant. All statistical analyses were performed using SPSS 16.0 (Chicago, IL, USA) and Graphpad Prism, version 5.0 (GraphPad Software Inc).

## Results

### Subject characteristics

Eighty patients and 80 age- and sex-matched controls were included for analysis. Baseline characteristics are shown in Table 1. Most patients were female (72.5%), with a mean age of 51 years. Consistent with previous reports, [24, 25] the main aetiology of bronchiectasis was idiopathic or post-infectious disease. Concomitant airway disease was common [14 (17.5%) and 5 (6.3%) had COPD and asthma, respectively]. Seventeen patients (21.3%) had PA colonization at baseline. No patients needed long-term oxygen therapy. Age, sex distribution and comorbidities did not differ between the two groups (all *P* > 0.05).

**Table 1:** Baseline characteristics of subjects with steady-state bronchiectasis and healthy controls

	Bronchiectasis	Controls	*P* values
Age, y	51.0 ± 13.9	49.5 ± 11.9	0.47
Male sex, No. (%)	22 (27.5)	22 (27.5)	1.00
Body-mass index, kg/m^2^	20.5 ± 2.0	25.2 ± 3.6	< 0.001
Current smoking, No. (%)	12 (15.0%)	18 (22.5%)	0.22
SBP, mmHg	120.9 ± 9.6	117.6 ± 11.2	0.62
DBP, mmHg	81.6 ± 8.6	73.4 ± 10.5	0.34
HR, bpm	82.6 ± 5.3	80.1 ± 4.2	0.001
Spirometry			
FVC% predicted	82.3 ± 16.7	–	–
FEV_1_% predicted	67.3 ± 21.4	–	–
mMRC grade	1.0 (0–2.0)	–	–
Exacerbation frequency in the past 12 mo	2.0 (1.3–5.0)	–	–
Hospital admission in the past 24 mo	2.0 (1.0–3.0)	–	–
Modiffied Reiff score for chest HRCT scan	5.0 (2.0–10.0)	–	–
PA colonization, No. (%)	17 (21.3%)	–	–
Aetiology			
Idiopathic, No. (%)	26 (32.5%)	–	–
Post infective, No. (%)	25 (31.3%)	–	–
COPD, No. (%)	14 (17.5%)	–	–
Asthma, No. (%)	5 (6.3%)	–	–
Others^a^, No. (%)	10 (12.5%)	–	–
BSI Scores			
Mild (0–4), No. (%)	27 (33.8%)	–	–
Moderate (5–8), No. (%)	11 (13.8%)	–	–
Severe (≥9), No. (%)	42 (52.5%)	–	–
Comorbidities			
Hypertension, No. (%)	1 (1.3%)	2 (2.5%)	0.56
Diabetes, No. (%)	6 (7.5%)	3 (3.8%)	0.30
Coronary heart disease, No. (%)	3 (3.8%)	3 (3.8%)	1.00
Stroke, No. (%)	0 (0%)	0 (0%)	…
Peripheral arterial disease, No. (%)	0 (0%)	0 (0%)	…
Clinical chemistry			
Cholesterol, mmol/L	3.8 ± 1.1	–	–
Triglyceride, mmol/L	1.0 ± 0.7	–	–
LDL-C, mmol/L	2.2 (1.9–2.4)	–	–
HDL-C, mmol/L	1.9 (1.0–1.4)	–	–
Systemic inflammatory markers			
IL-6, pg/mL	3.3 (1.8–7.9)	–	–
IL-8, pg/mL	7.1 (4.3–11.8)	–	–
C-reactive protein, mg/dL	1.17 (0.3–3.9)	–	–
Fibrinogen, g/L	3.2 ± 0.9	–	–

Data are depicted as mean ± SD, median (interquartile range [IQR]) or n (%)

*BSI* bronchiectasis severity index, *COPD* chronic obstructive pulmonary disease, *DBP* diastolic blood pressure, *FEV*_*1*_ forced expiratory volume in one second, *FVC* forced vital capacity, *HR* heart rate, *HRCT* high-resolution computed tomography, *IL* interleukin, *mo* month, *mMRC* modified Medical Research Council, *PA Pseudomonas aeruginosa*, *SBP* systolic blood pressure; y = year

^a^Aspiration (*n* = 2), Allergic Bronchopulmonary Aspergillosis (*n* = 3), Microscopic Polyangiitis (*n* = 1), Rheumatoid Arthritis (*n* = 1), Inflammatory Bowel Disease (*n* = 1), Kartagener’s Syndrome (*n* = 1), Primary Immunodeficiency (*n* = 1)

### Reproducibility of baPWV in bronchiectasis

**Fig. 1:**
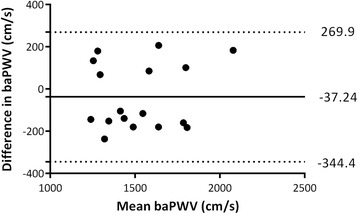
Reproducibility of the baPWV. Bland-Altman plot of the baPWV over 2 weeks in 17 patients with steady-state bronchiectasis whose treatment remained unchanged. Solid line represents the mean difference of baPWV between the baseline and 2-week interval (− 37.24); Dotted line represents 95% limits of agreement (− 344.4 to 269.9)

The mean ± SD baPWV of 17 bronchiectasis patients who had baPWV measurement at baseline and 2-week interval were 1507.18 ± 264.04 cm/s and 1544.41 ± 240.71 cm/s, respectively (*P* = 0.67). The Bland-Altman plot of baPWV is shown in Fig. 1; the difference of baPWV was − 37.24 (156.70) cm/s. The Pearson correlation coefficient was 0.81 (*P* < 0.001) and intra-class correlation coefficient for baPWV was 0.89 (0.71–0.96), suggesting a good reproducibility.

### Arterial stiffness assessment

**Fig. 2:**
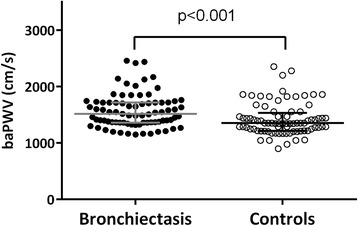
The median (IQR) of baPWV in patients with steady-state bronchiectasis [1514 (1362–1718) cm/s] and healthy controls [1352 (1218–1535) cm/s] (*P < 0.001 by Mann-Whitney test). Significantly higher baPWV was found in bronchiectasis patients compared with healthy controls

**Fig. 3:**
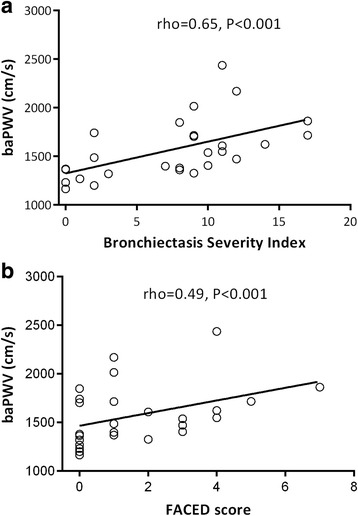
The relationship between baPWV and bronchiectasis severity assessed with BSI (rho = 0.65, *P* < 0.001 by Spearman’s correlation analysis; (**a**) and FACED scores (rho = 0.49, *P* < 0.001 by Spearman’s correlation analysis; (**b**). BaPWV significantly correlated with BSI and FACED scores

Patients with bronchiectasis had significantly higher baPWV than control subjects [median (IQR); 1514 (1362–1718) cm/s vs. 1352 (1218–1535) cm/s, *P* < 0.001] (Fig. 2). Subgroup analysis by excluding subjects with hypertension, coronary heart disease or diabetes demonstrated similar findings, with bronchiectasis patients (*n* = 70) having higher baPWV (*P* < 0.001) than control subjects (*n* = 72) (Additional file 1: Figure S1). The level of baPWV when stratified by PA colonization is shown in Additional file 1: Figure S2. Patients with PA colonization had significantly higher baPWV compared with those without [median (IQR); 1718 (1617–1864) cm/s vs. 1405 (1328–1705) cm/s, *P* < 0.001]. Moreover, both right and left baPWV values were markedly higher in bronchiectasis patients than in control subjects (both *P* < 0.001; Additional file 1: Table S1). To explore the impacts of disease severity on arterial stiffness, bronchiectasis severity was assessed with both BSI and FACED scores. We observed a significant correlation between baPWV and BSI score (rho = 0.65, *P* < 0.001; Fig. 3a). Similar association was also applied for baPWV and FACED score (rho = 0.49, *P* < 0.001; Fig. 3b). Notably, there was no significant correlation between systemic inflammatory markers [i.e. C-reactive protein (CRP), interleukin-6 (IL-6), IL-8 and fibrinogen] and the BSI (data not shown, all *P* > 0.05).

### Determinants of baPWV in bronchiectasis

In univariable analysis, factors that had a significant effect on baPWV included age, BMI, smoking history, SBP, FEV_1_% predicted, FVC% predicted, exacerbation frequency within 1 year, hospitalization frequency within 2 years, PA colonization, MRC dyspnea score, HRCT score and fibrinogen levels (Table 2). In multivariable analysis, age (β = 0.348, *P* < 0.001), PA colonization (β = 0.282, *P* < 0.001), SBP (β = 0.257, *P* = 0.002), BMI (β = 0.250, *P* = 0.001) and exacerbation frequency within one year (β = 0.195, *P* = 0.07), but not systemic inflammatory markers, were independent factors influencing on the baPWV in bronchiectasis (Table 3).

**Table 2:** Results of univariable regression analysis for baPWV of bronchiectasis patients

Variable	β	*P* values
Anthropometry		
Age	0.747	< 0.001
Male Sex	0.214	0.056
BMI (kg/m^2^)	0.384	< 0.001
Smoking	0.248	0.026
SBP, mmHg	0.617	< 0.001
DBP, mmHg	0.177	0.116
HR, bpm	0.108	0.341
Lung Function		
FEV_1_% predicted	−0.468	< 0.001
FVC% predicted	−0.478	< 0.001
Disease-related parameters		
No. of exacerbations within 12 mo	0.289	0.009
No. of hospitalization within 24 mo	0.499	< 0.001
PA colonization	0.442	< 0.001
Other bacterial colonization	−0.103	0.365
MRC dyspnea score	0.249	0.026
HRCT score	0.405	< 0.001
Comorbidities		
COPD	0.189	0.093
Coronary Heart Disease	0.042	0.709
Diabetes	0.217	0.054
Laboratory-related parameters		
Cholesterol, mmol/L	0.115	0.310
Triglyceride, mmol/L	−0.002	0.984
LDL-C, mmol/L	0.105	0.355
HDL-C, mmol/L	−0.113	0.316
IL-6, pg/mL	0.120	0.300
IL-8, pg/mL	0.150	0.170
C-reactive protein, mg/dL	0.170	0.130
Fibrinogen, g/L	0.249	0.026

*BaPWV* brachial ankle pulse-wave velocity, *BMI* body-mass index, *COPD* chronic obstructive pulmonary disease, *DBP* diastolic blood pressure, *FEV*_*1*_ forced expiratory volume in one second, *FVC* forced vital capacity, *HR* heart rate, *HRCT* high-resolution computed tomography, *IL* interleukin, *mMRC* modified Medical Research Council, *mo* month, *No* number, *SBP* systolic blood pressure, *PA* Pseudomonas aeruginosa, *y* year

**Table 3:** Results of multiple regression analysis for baPWV of bronchiectasis patients

Variable	β	P
Age	0.348	< 0.001
PA colonization	0.282	< 0.001
SBP, mmHg	0.257	0.002
BMI (kg/m^2^)	0.250	0.001
No. of exacerbations within 1 year	0.195	0.007

*BaPWV* brachial ankle pulse-wave velocity, *BMI* body-mass index, *PA Pseudomonas aeruginosa*, *SBP* systolic blood pressure

## Discussion

Our study found that bronchiectasis patients have increased baPWV compared with healthy controls, which was validated in subgroup analysis excluding individuals with concomitant hypertension or coronary heart disease or diabetes. BaPWV correlated with disease severity assessed with both BSI and FACED scores. Aging, PA colonization, SBP, BMI and exacerbation frequency in the previous year, but not systemic inflammatory markers, were independent factors affecting the baPWV in patient with bronchiectasis.

Recent studies have demonstrated that bronchiectasis might be an independent risk factor for CVD, which was unrelated to smoking--traditional cardiovascular risk factors or comorbidities associated with the etiology of bronchiectasis. This highlighted the importance of preventing from further development of CVD in bronchiectasis [3–5, 7]. Thus, identifying the early changes of cardiovascular system before the occurrence of major clinical events (i.e. stroke and myocardial infarction), have additional implications for CVD prevention at individual levels, such as early-stage targeted prevention and intervention for high-risk patients. Arterial stiffness has a strong predictive value for cardiovascular events beyond traditional cardiovascular risk factors in healthy populations [9], as well as in patients with chronic lung diseases, and is the most suited parameter for routine clinical practice. Prior to our study, only one case-control study has reported that patient with bronchiectasis (*n* = 20) had increased arterial stiffness, measured by aortic PWV, compared with matched controls [18]. Similar results were also validated in our study despite using different indicators of arterial stiffness. We have, for the first time, demonstrated that baPWV correlated positively with the disease severity which was assessed with both the BSI and FACED scores, the two-validated composite disease severity metrics, suggesting that arterial stiffness developed early in bronchiectasis and aggravates along with greater disease severity or disease progression. A meta-analysis has shown that an increase in baPWV by 1 m/s corresponded to an increase of 12 and 13% in the total cardiovascular events and cardiovascular mortality, respectively [27]. The elevation in baPWV between bronchiectasis patients and control subjects in our cohort was 1.62 m/s, which might result in an estimated increase of 19 and 21% in total cardiovascular events and cardiovascular mortality, respectively. In this regard, this difference should be sufficient to elicit adverse outcomes in patients with bronchiectasis.

Global assessments of bronchiectasis severity with both BSI and FACED scores showed positive correlation with baPWV. This was further confirmed in our multivariate analysis that aging, PA colonization and the number of exacerbation within one year (all crucial components of the BSI) were independent factors influencing on baPWV in bronchiectasis. Recently, Evans et al. [5] have reported that bronchiectasis severity was independently associated with vascular disease, which was also consistent with our findings. Interestingly, the average age of bronchiectasis patients in our cohort was 51 years, which was significantly lower than that in European and US cohorts (70 years and 64 years, respectively) [28, 29]. Since the fact that age was the strongest predictive factor of baPWV in our cohort, age-related changes in baPWV would be more prominent in European and US bronchiectasis cohorts, which merits further validations. Meanwhile, we found that PA colonization and the number of exacerbation within one year were significantly associated with arterial stiffness. The median difference of baPWV in patients with PA colonization compared to those without was 3.13 m/s (unadjusted analysis), which corresponded to a 38% increase in excess risks of cardiovascular events [27]. Arterial stiffness was reportedly increased in cystic fibrosis children colonized with PA compared with uninfected children [30], which was consistent with our findings. PA colonization may represent a distinct clinical phenotype of bronchiectasis with poorer quality-of-life, frequent exacerbation and poorer long-term outcomes [31–33]. Our findings have extended the rationale to optimize management of patients with PA colonization, by integrating the concept of reduction in cardiovascular events. The association between exacerbation frequency and cardiovascular risk has been reported in COPD, and that chronic low-grade systemic inflammation might be one of the possible mechanisms linking frequent exacerbations to increased arterial stiffness [34]. However, the lack of association between systemic inflammatory markers and arterial stiffness in our study indicated that this assumption cannot be extrapolated to bronchiectasis patients. Nevertheless, further studies are merited to investigate the links between chronic bacterial infection, exacerbation, systemic inflammation and increased arterial stiffness by inclusion of greater sample sizes and longer follow-up duration.

Systemic inflammation has been proposed to accelerate and stimulate vascular extracellular matrix remodeling process of elastin fragmentation and collagen deposition, resulting in increased arterial stiffness [35]. However, data for the causal link between low-grade systemic inflammation and a higher incidence of cardiovascular disease in bronchiectasis are lacking [5]. In this study, although we found an association between baPWV and plasma fibrinogen, but not IL-6, IL-8 and CRP in univariate regression models, the results could not be further confirmed in multivariate model. Furthermore, systemic inflammatory markers did not correlate with bronchiectasis severity as assessed with BSI. However, we could not preclude the possibility that other known or unidentified inflammatory markers (i.e. intercellular adhesion molecule-1, vascular cell adhesion molecule, and E-selectin) might be related to arterial stiffness. Saleh et al. [36] recently shows that bronchiectasis patients presented with significantly heterogeneous levels of systemic inflammatory proteins, which could not be adequately accounted for by the differences in disease aetiology or severity. This indicated that persistent systemic inflammation may affect only some bronchiectasis patients as evidenced in COPD [37]. To further answer this question, it would be helpful to investigate exclusively in this specific subgroup of patients whether the correlation between inflammatory markers and increased arterial stiffness exists.

In addition, changes of arterial stiffness in bronchiectasis patients could not be explained by traditional cardiovascular risk factors, such as plasma lipids [38], diabetes [39], smoking history [40] and FEV_1_ [41], all powerful risk factors for predicting arterial stiffening. This might be because of a lack of effect of these risk factors per se, or because arterial stiffness was not driven by an atherosclerotic process at least in its early stages but rather by an alternative pathologic factor in which blood pressure plays a role [42]. It is well recognized that arterial stiffness depends on mechanical stretch of the arterial wall and, hence, on blood pressure at the time of the measurement [43]. Stretch is thought to transfer loading to stiffer elements within the wall that are of greater tensile strength (i.e. from elastin to collagen) and, therefore results in an overall stiffening of the wall. Accordingly, systolic blood pressure was independently associated with greater arterial stiffness in our study.

A major strength is that we have conducted the reproducibility of baPWV measurement and, for the first time, systematically investigated arterial stiffness in bronchiectasis patients. However, several limitations merit interpretation. First, the study design was cross-sectional, and clinical follow-up data were not available, which precluded the causality inference between bronchiectasis, bacterial colonization, systemic inflammation, arterial stiffness and CVD, and the direct comparison of predictive value of baPWV and cardiovascular risk scores for future cardiovascular events, which merits further investigation. Second, the inclusion of patients and controls subjects with CHD, diabetes and hypertension might be criticized, although subgroup analysis with exclusion of these patients did not alter the findings. Thirdly, there are still some debates regarding whether COPD and asthma should be viewed as aetiologies of bronchiectasis [44], and abundant evidence of elevated arterial stiffness has been reported in COPD [14, 15]. We therefore conducted an exploratory subgroup analysis by removing patients with COPD or asthma in order to determine whether inclusion of these patients might have biased our findings. Reassuringly, baPWV remained significantly greater in bronchiectasis patients than those healthy controls (median; 1488 cm/s versus 1352 cm/s, *P* = 0.004). Finally, left ventricular morphology and function were not assessed, which have been reportedly associated with arterial stiffness [45].

## Conclusions

Patients with bronchiectasis had significantly higher baPWV compared with control subjects, mainly determined by age, PA colonization, SBP, BMI and the number of exacerbation in the last year. Disease severity, but not systemic inflammatory markers, was associated with the degree of baPWV. Further studies are needed to determine the usefulness of PWV measurement in risk stratification and clinical management of bronchiectasis patient to optimize cardiovascular outcomes.

## Additional file

Additional file 1: **Figure S1.** The median (IQR) of baPWV in patients with steady-state bronchiectasis (*n* = 70) and healthy controls (*n* = 72) when excluded subjects with hypertension, coronary heart disease or diabetes (*p < 0.001 by Mann-Whitney test). Significantly higher baPWV was found in bronchiectasis patients compared to healthy controls. **Figure S2.** The median (IQR) of baPWV in patients with steady-state bronchiectasis stratified by PA colonization (*p < 0.001 by Mann-Whitney test). Significantly higher baPWV was found in bronchiectasis patients with PA colonization than those without. **Table S1.** Right and left baPWV in patients with steady-state bronchiectasis and healthy controls. (ZIP 57 kb)

## Abbreviations

BaPWV: Brachial-ankle pulse wave velocity
BMI: Body-mass index
BSI: Bronchiectasis Severity Index
cfPWV: Carotid-femoral pulse-wave velocity
COPD: Chronic obstructive pulmonary disease
CVD: Cardiovascular disease
DBP: Diastolic blood pressure
FEV1: Forced expiratory volume in one second
FVC: Forced vital capacity
HRCT: High-resolution computed tomography
IL: Interleukin
IQR: Interquartile range
PA: Pseudomonas aeruginosa
PPMs: Potentially pathogenic microorganisms
PWV: Pulse-wave velocity
SBP: Systolic blood pressure
SD: Standard deviation
