# Supplementary material for: Arterial stiffness in adults with steady-state bronchiectasis: association with clinical indices and disease severity
Source: Respir Res. 2018 May 9;19:86. doi: 10.1186/s12931-018-0790-3 (PMC5944117; doi:10.1186/s12931-018-0790-3)
Supplement: Supplementary file 1 — Figure S1. The median (IQR) of baPWV in patients with steady-state bronchiectasis (n = 70) and healthy controls (n = 72) when excluded subjects with hypertension, coronary heart disease or diabetes (*p < 0.001 by Mann-Whitney test). Significantly higher baPWV was found in bronchiectasis patients compared to healthy controls. Figure S2. The median (IQR) of baPWV in patients with steady-state bronchiectasis stratified by PA colonization (*p < 0.001 by Mann-Whitney test). Significantly higher baPWV was found in bronchiectasis patients with PA colonization than those without. Table S1. Right and left baPWV in patients with steady-state bronchiectasis and healthy controls. (ZIP 57 kb) [file 12931_2018_790_MOESM1_ESM.zip › Online Supplement.docx]

| **Table S. Right and left baPWV in patients with steady-state bronchiectasis and healthy controls** | | | |
| --- | --- | --- | --- |
|  | **Bronchiectasis** | **Controls** | **P value** |
| Right baPWV | 1524 (1349-1803) | 1382 (1223-1569) | <0.0001 |
| Left baPWV | 1497 (1358-1682) | 1351 (1220-1515) | 0.0009 |

**Figure legends**

**Figure S1** The median (IQR) of baPWV in patients with steady-state bronchiectasis (n=70) and healthy controls (n=72) when excluded subjects with hypertension, coronary heart disease or diabetes (*p<0.001 by Mann-Whitney test). Significantly higher baPWV was found in bronchiectasis patients compared to healthy controls.

**Figure S2** The median (IQR) of baPWV in patients with steady-state bronchiectasis stratified by PA colonization (*p<0.001 by Mann-Whitney test). Significantly higher baPWV was found in bronchiectasis patients with PA colonization than those without.
